# Predicting the risk of mortality and rehospitalization in heart failure patients: A retrospective cohort study by machine learning approach

**DOI:** 10.1002/clc.24239

**Published:** 2024-02-25

**Authors:** Marzieh Ketabi, Aref Andishgar, Zhila Fereidouni, Maryam Mojarrad Sani, Ashkan Abdollahi, Mohebat Vali, Abdulhakim Alkamel, Reza Tabrizi

**Affiliations:** ^1^ Student Research Committee Fasa University of Medical Sciences Fasa Iran; ^2^ USERN Office Fasa University of Medical Sciences Fasa Iran; ^3^ Department of Medical Surgical Nursing Fasa University of Medical Science Fars Iran; ^4^ School of Medicine Tehran University of Medical Sciences Tehran Iran; ^5^ School of Medicine Shiraz University of Medical Sciences Shiraz Iran; ^6^ Student Research Committee Shiraz University of Medical Sciences Shiraz Iran; ^7^ Noncommunicable Diseases Research Center Fasa University of Medical Science Fasa Iran; ^8^ Clinical Research Development Unit Fasa University of Medical Sciences Fasa Iran

**Keywords:** cohort studies, heart failure, machine learning, mortality, patient readmission

## Abstract

**Background:**

Heart failure (HF) is a global problem, affecting more than 26 million people worldwide. This study evaluated the performance of 10 machine learning (ML) algorithms and chose the best algorithm to predict mortality and readmission of HF patients by using The Fasa Registry on Systolic HF (FaRSH) database.

**Hypothesis:**

ML algorithms may better identify patients at increased risk of HF readmission or death with demographic and clinical data.

**Methods:**

Through comprehensive evaluation, the best‐performing model was used for prediction. Finally, all the trained models were applied to the test data, which included 20% of the total data. For the final evaluation and comparison of the models, five metrics were used: accuracy, F1‐score, sensitivity, specificity and Area Under Curve (AUC).

**Results:**

Ten ML algorithms were evaluated. The CatBoost (CAT) algorithm uses a series of decision tree models to create a nonlinear model, and this CAT algorithm performed the best of the 10 models studied. According to the three final outcomes from this study, which involved 2488 participants, 366 (14.7%) of the patients were readmitted to the hospital, 97 (3.9%) of the patients died within 1 month of the follow‐up, and 342 (13.7%) of the patients died within 1 year of the follow‐up. The most significant variables to predict the events were length of stay in the hospital, hemoglobin level, and family history of MI.

**Conclusions:**

The ML‐based risk stratification tool was able to assess the risk of 5‐year all‐cause mortality and readmission in patients with HF. ML could provide an explicit explanation of individualized risk prediction and give physicians an intuitive understanding of the influence of critical features in the model.

## Highlight

- Length of stay in the hospital was the most important predictor of hospital readmission.
- Hemoglobin level was the most critical predictor of 1‐month mortality.
- Family history of Mi was the most important predictor of 1‐year mortality.
- Catboost algorithm performed the best of the ten models studied.

## Graphical abstract

We evaluated ten ML algorithm to predict mortality and readmission of HF patients by using (FaRSH) database. For the final evaluation and comparison of the models, five metrics were used: accuracy, F1‐score, sensitivity, specificity and Area Under Curve(AUC). Finally Tthe CatBoost algorithm performed the best. The most significant variables to predict the events were length of stay in the hospital, hemoglobin level and family history of MI.

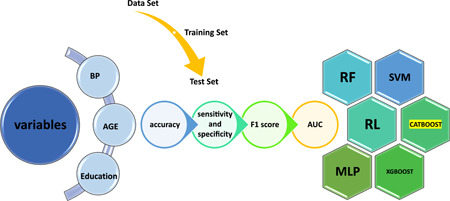

## 1 INTRODUCTION

Heart failure (HF) is a global problem, affecting more than 26 million people worldwide.^1^ In the United States, over 1 million people are hospitalized due to HF yearly.^2^, ^3^ In Iran, results from the Persian Registry of Cardiovascular Disease/heart failure (PROVE/HF) study in Isfahan province showed that the annual rate of readmission and mortality were high.^4^ For these patients, readmission or death in the postdischarge phase was problematic. In Europe, the mortality rate is 7% within the year and increases to 26.7% each year after hospitalization for HF,^5^ and readmission rate within the year is between 20% and 25%.^6^, ^7^ Approximately 30% of HF patients may be readmitted within 30–60 days after discharge.^8^, ^9^, ^10^, ^11^ HF enforces a giant economic burden. Global costs associated with HF are projected to rise to approximately $400 billion by 2030.^12^

Although advances in medical and percutaneous therapies such as percutaneous coronary intervention (PCI), angiography, echocardiography, and low high‐density lipoprotein (HDL) cholesterol,^13^ copeptin,^14^ and B‐type natriuretic peptide^15^ factors have been used to predict HF,^16^, ^17^ HF patients are vulnerable to hospital readmission, high mortality, critical damage to the quality of life, and results in significant financial stress on the public health‐care system.^17^, ^18^

In recent years, artificial intelligence has increasingly penetrated the medical field to detect diseases.^19^ Identifying disease patterns by processing information in artificial intelligence can help provide better healthcare to patients.^19^ Confidence in using machine learning algorithms has increased in the health sector due to their ability to consider complex relationships between clinical parameters using complex mathematical formulations.^20^

ML algorithms are used in many fields of medicine, such as diagnosis, prediction, treatment, and explanation of medical images.^21^, ^22^ The ML algorithm informs the physicians and patients about the prognosis of the disease, decision‐making, disease management, end‐of‐life preferences and increases the motivation of patients to follow the treatment.^23^

Li et al. compared the performance of the XG Boost prediction model with three other models by examining 2098 intensive care unit (ICU) patients in a retrospective cohort study. Finally, the XG Boost model had the highest prediction performance among the four models, with an AUC of 0.824. In contrast, SVM had the weakest ability, and the average blood urea nitrogen (BUN) was recognized as the most critical predictor variable.^24^ A study by Peng et al. aimed to select a ML algorithm to predict 28‐day mortality in HF patients with hypertension in the ICU. Neural network (NN) models had the best predictive performance in the test set and external validation cohort, respectively, outperforming traditional logistic regression analysis.^25^ Angraal et al. conducted a study to predict mortality and readmission of HF patients with preserved ejection fraction (HFPEF). They used Logistic Regression (LR), Random Forest (RF), gradient descent, and SVM models during 3 years of follow‐up. The RF model had the best performance for predicting mortality with AUC of 0.72 and readmission with AUC of 0.76.^23^

Considering the conflicting results in the available evidence and limited studies in predicting all the consequences of HF patients, we decided to conduct a study for the first time in southeastern Iran aiming at predicting the mortality and readmission of HF patients using the latest machine learning algorithms based on registry data and examining the outcomes of HF.

## 2 METHODS

### 2.1 Study design

This retrospective cohort study used the records from the Fasa Registry on Systolic Heart Failure (FaRSH). Fasa, a city of around 250,000 inhabitants, is located in Fars province in southwest Iran. This study gathered data from the people of Fasa and 34 surrounding towns and villages. This research included participants hospitalized due to HF, whether they were acute new‐onset HF or individuals diagnosed with acute decompensation of chronic HF.

### 2.2 Data source

We used the FaRSH database, which consists of 2488 patients with HF. All patients with the census method were included. The research was meant to provide a 1‐year follow‐up for each participant with a recruiting period from March 2015 to March 2020, with the follow‐up ending in March 2025. All people included in the registry were evaluated based on their admission and discharge diagnosis of systolic HF, as determined by the attending cardiologists who examined patients daily and used the International Classification of Diseases, Tenth Revision (ICD‐10) coding (Supporting Information S1: Table 1).^26^

### 2.3 Outcomes

After being admitted to the hospital and having their information documented, the patients were followed for 1 month and 1 year, gathered into our three outcomes: 1‐month mortality, 1‐year mortality, and hospital readmission, respectively. The outcomes were analyzed as dummy variables. The first two outcomes were given as dead or living, whereas the third was hospitalization or not for the second time due to HF.

### 2.4 Predictors

Fifty‐seven factors were evaluated as independent variables to predict outcomes based on a literature review and clinical relevance connected to HF. All these factors were entered into and assessed using machine learning algorithms. Meanwhile, in machine learning, several continuous variables were classified to boost computation speed, known as discretization.^27^

Two nurses were exceptionally trained for patient evaluation and data entry. For at least 1 month, the nurses were taught by a single supervising senior cardiology nurse and five collaborating cardiologists. They documented information on patients at the time of their admission.

Patients' demographic and anthropometric information, such as age, gender, ethnicity, marital status, place of residence, waist circumference, and body mass index (BMI), were obtained. People were separated into three groups based on ethnicity: Arabs, Persians, and others. They were split into two categories based on their marital status: married and unmarried. People were sorted into three categories based on their BMI: normal (<18.5), overweight (18.5–24.9), and obese (>25).^23^

The waist circumference cut‐off values for abdominal obesity in males and females were 102 and 88 cm, respectively.^28^, ^29^ They were asked about smoking cigarette and their opium usage. The patients were divided into four groups based on the New York Heart Association (NYHA) classification. In addition, information about the participants' underlying disorders and diseases throughout their hospitalization was gathered. The conditions examined as factors were dilated cardiomyopathy, right ventricular (RV) failure, previous myocardial infarction (MI), atrial fibrillation/flutter (AF), chronic obstructive pulmonary disease (COPD), heart valve disease, prior stroke, left bundle branch block (LBBB), hypertension, and diabetes. In addition, patients were split into three groups depending on previous chronic HF hospitalization: not hospitalized, hospitalized less than 30 days ago, and hospitalized more than 30 days ago. Finally, they were asked about the duration of their HF, which was divided into two categories: over 6 months and below 6 months. The participants' medical histories were fully gathered before and after their hospitalization. This study's desired therapeutic factors were angiotensin‐converting enzyme (ACE) inhibitors, β‐blockers, mineralocorticoid receptor antagonists (MRAs), diuretics, digitalis, statins, long‐acting nitrates, anticoagulants, intravenous inotropic, and acetylsalicylic acid (ASA) or antiplatelet. Information on device therapy in patients was collected, and they were separated into four groups, including pacemaker, implantable cardioverter‐defibrillator (ICD), cardiac resynchronization therapy defibrillator (CRT‐D), and having no device. In addition, they provided information about invasive procedures such as coronary artery bypass graft (CABG) and percutaneous coronary intervention (PCI). During hospitalization, blood samples were taken, and levels of hemoglobin, cholesterol, triglyceride, high‐density lipoprotein cholesterol (HDL‐C), low‐density lipoprotein cholesterol (LDL‐C), sodium level, potassium level, random blood sugar, white blood cell (WBC), and creatinine were retrieved. The Cockcroft–Gault formula was used to calculate glomerular filtration rate (GFR). The cut‐off hemoglobin level was set at 13 for males and 12 for females.^30^ The cholesterol, triglyceride, and LDL threshold values were 200, 150, and 130, respectively. HDL < 50 (female) or HDL < 40 (male) was considered abnormally low levels.^30^ The patients were separated into three groups based on their sodium and potassium levels: normal, hypo, and hyper. The remaining laboratory variables were continually entered into the devices. Based on their heart rate (HR), patients were categorized into three groups: bradycardia (HR < 60), normocardia (60 ≤ HR ≤ 100), and tachycardia (100 < HR). The variables of systolic and diastolic blood pressure were evaluated quantitatively. The patients were asked about stroke, HF, and MI in their first‐degree relatives. Depending on their caregivers, people were separated into two hospital and clinical care categories. During their stay, patients' electrocardiograms (ECGs) were collected and classified into one of four categories: sinus rhythm, pacemaker rhythm, atrial fibrillation, and rhythms. Transthoracic echocardiography was done on the patients throughout their hospitalization. They were separated into four groups, which included normal range (50% ≤ left ventricular ejection fraction [LVEF] < 70%), mild dysfunction (40 ≤ LVEF ≤ 49), moderate dysfunction (30 ≤ LVEF ≤ 39), and severe dysfunction (LVEF < 30).^31^ Finally, when the patients were discharged, their hospital stay was recorded.

### 2.5 Data cleaning

SPSS software version 18 (IBM Corp.) was utilized to evaluate the available data in this investigation. There was less than 15% missing data in all variables. The missing data were replaced using multiple imputations (MI) with an automatic method. MI is a predictive approach for handling missing data in multivariate analysis. MI combines classical and Bayesian statistical techniques and uses specific iterative algorithms to generate multiple imputations. MI aims to provide acceptable estimates of missing values, accurately reflect uncertainty, and preserve important data relationships and aspects of the data distribution. MI requires the analyst to specify an imputation model, input multiple data sets, analyze them separately, and then combine the results. MI yields a single set of test statistics, parameter estimates, and standard errors. A descriptive analysis table of variables was also created in SPSS. *p* values less than .05 were considered statistically significant.

### 2.6 Normalization

The data should be modified to minimize computations and improve model accuracy. Two approaches feature scaling and one‐hot encoding were employed in this procedure. The first approach transformed the values of all continuous variables to a range of −1 to +1. For variables with more than two categories, the second technique was utilized, and categorical variables were transformed into numerical variables consisting of zeros and ones.

### 2.7 Splitting total data

All data were first split into training and test sets. Test data contained 20% of the entire data. The machine algorithm utilized the training set to teach itself, while the testing set was used to assess the classifier's prediction error rate after learning.

### 2.8 Feature selection

**Figure 1:**
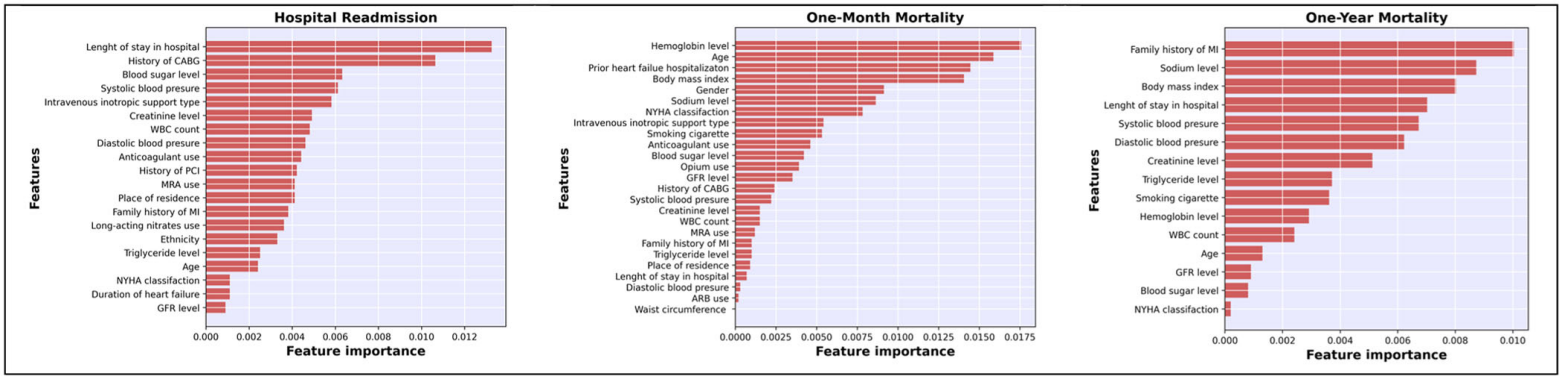
Top variable importance values for predicting hospital readmission, 1‐month mortality, and 1‐year mortality in heart failure patients using Cat boost (CAT). ARB, angiotensin receptor blockers; CABG, coronary artery bypass graft; COPD, chronic obstructive pulmonary disease; GFR, glomerular filtration rate; LBBB, left bundle branch block; MI, myocardial infarction; MRA, mineralocorticoid receptor antagonist; NYHA classification, New York Heart Association Classification; PCI, percutaneous coronary intervention; WBC, white blood count.

Feature selection approaches play a crucial role in achieving efficient data reduction, which is essential for developing accurate models. In this work, we employed recursive feature elimination (RFE) in conjunction with a tree‐based machine learning model to implement a wrapper technique. RFE is an iterative process that involves repeatedly training a machine learning model and removing the lowest‐ranking features, ultimately identifying the most relevant predictors. By utilizing RFE, we were able to identify the ideal number of variables and their combinations for predicting hospital readmission (20 variables), 1‐month mortality (25 variables), and 1‐year mortality (15 variables). Figure 1 illustrates the top predictors for each outcome.

## 3 MACHINE LEARNING MODELS

In this study, 10 supervised machine learning algorithms were utilized, which comprised LR, decision tree (DT), SVM, RF, Gaussian Naive Bayes (GNB), linear discriminant analysis (LDA), K‐nearest neighbors (KNN), gradient maximization (GBM), XGBoost (XGB), and CAT Boost (CAT). These ML models use a variety of methodologies and complicated calculations. Depending on the data type, they can carry out various functions. As a result, by applying many models, we may eventually identify the optimum model for our data. Anaconda (Version 4.12.0) implemented all ML algorithms on the Jupyter Notebook Platform (Version 3.3.2). Furthermore, the machine algorithms were run using the Scikit‐Learn Module (Version 1.1.3).

Logistic regression: Logistic regression is a traditional machine learning method used to predict the probability of an event occurring. This method creates a linear model on the training data by using a logistic function to calculate the probability of an event.

Decision tree: A decision tree is a machine learning method used to classify data. This method creates a nonlinear model on the training data using a series of rules.

Support vector machine: SVM is a machine learning technique used to classify data. This method creates a nonlinear model on the training data by using a boundary line to separate two classes of data.

Random forest: Random forest is a machine learning method that uses a set of decision trees to create a nonlinear model.

Gaussian Naive Bayes: Gaussian Naive Bayes is a machine learning method that uses a Gaussian probability distribution to calculate the probability of an event occurring.

Linear discriminant analysis: Linear discriminant analysis (LDA) is a machine learning method used to classify data. This method creates a model on the training data by using a linear model to separate two classes of data.

K‐nearest neighbors: K‐nearest neighbors (KNN) is a machine learning method used to classify data. This method builds a model on the training data using the K samples closest to the new sample.

Gradient maximization: Gradient maximization (GBM) is a machine learning method that uses a series of decision tree models to create a nonlinear model.

XGBoost: XGBoost is a machine learning method that uses a series of decision tree models to build a nonlinear model.

CatBoost: CatBoost is a machine learning method that uses a series of decision tree models to create a nonlinear model.

We were interested in comparing the performance of these machine learning techniques in predicting the prognosis of HF. These techniques use a wide variety of algorithms and approaches, so we believe that this comparison can provide valuable insight into the best technique for this task.

### 3.1 Data augmentation and hyperparameter tuning

When dealing with asymmetric data sets, data imbalance is a problem. For example, the data set would be imbalanced, if categorical outcomes were distributed unequally. According to the three final results of this study, which included 2488 patients, only 366 (14.7%) were readmitted to the hospital, 97 (3.9%) died within a month of the follow‐up, and 342 (13.7%) died within a year of the follow‐up. To protect the machine learning process, the ratio of our output values should be shared equally. If this is not done, the models may be more accurate, but the F1 score for predicting death and rehospitalization will likely be much lower, and we will not achieve the primary aim of this study.^32^ Oversampling was utilized to balance the values of each outcome, and data with the outcomes of death and rehospitalization were obtained. The Synthetic Minority Oversampling Technique (SMOTE) is one of the most remarkable oversampling techniques. In this method, the minority class is oversampled by creating “synthetic” instances rather than oversampling using replacement. SMOTE selects samples from the minority class and creates “fake” samples along the same line segment, linking some or all of the minority class's KNN.^33^

The fivefold cross‐validation and hyperparameter tuning approaches were used to train the models and determine the optimal values for each model. Both techniques were only utilized on training data. All of the training data was divided into five equal parts in the fivefold approach, and each time one of the parts was considered validation data, it trained itself. It reported the accuracy, and lastly, the average of all five accuracies was obtained. Each machine's accuracy may now be altered by adjusting its hyperparameters. A different variety of hyperparameters was employed in the hyperparameter tuning procedure.^34^ To find the optimal combination of hyperparameters.

Data leakage is an issue that frequently arises when SMOTE is used; thus, care should be taken while using SMOTE through a hyperparameter tuning procedure. Only four more components in each fold of the hyperparameter were augmented, not the validation data.

### 3.2 Data augmentation and training the machine learning algorithms

Models were trained using the training data after the best hyperparameter combinations for each model were identified. In addition, 80% of the total data was in the training set, which is unbalanced. To help the models be trained more effectively, the training data set was augmented and transformed into a balanced data set. Moreover, 20% of the total data was in the test set, and the models had never seen this data before. Test data is a helpful data set for assessing models since it is unbalanced like real‐world data.

### 3.3 Model evaluation

Finally, all the trained models were applied to the test data, including 20% of the total data. Five metrics were used for the final evaluation and comparison of the models: accuracy, sensitivity, specificity, F1 score, and AUC. The following equations are used to determine the evaluation metrics:

(1) Accuracy=(TP+TN)/(TP+FP+TN+FN),

(2) Sensitivity=TP/(TP+FN),

(3) Specificity=TN/(TN+FP),

(4) $$F1\text{score}\;=2\times \frac{\mathrm{TP}}{2\times \mathrm{TP}+\mathrm{FP}+\mathrm{FN}},$$

where TP is the true positive rate, TN is the true negative rate, FP is the false positive rate, and FN is the false negative rate. AUC and F1 score are the ideal metrics to use to choose the optimal ML model due to the imbalanced structure of the data.

### 3.4 Feature importance

The model with the greatest performance for all three outcomes was used to rank the variables, and the top predictive variables for each outcome were displayed in order. However, the one‐by‐one process of machine learning is summarized in Supporting Information S1: Figure 1.

### 3.5 Ethics declarations

The study protocol was approved by the Research Council and the Ethics Committee of Fasa University of Medical Sciences (IR.FUMS.REC.1401.113) and all methods were performed in accordance with the relevant guidelines and regulations to this effect. Informed consent was obtained from all subjects and/or their legal guardian(s).

## 4 RESULTS

### 4.1 Basic characteristics of participants

According to the three outcomes from this study, which involved 2488 participants, 366 (14.7%) of the patients were readmitted to the hospital, 97 (3.9%) of the patients died within a month of the follow‐up, and 342 (13.7%) of the patients died within a year of the follow‐up. Supporting Information S1: Tables 2–4 give a descriptive analysis of variables according to the three planned outcomes. Table 1 displays descriptive characteristics of variables without respect to outcomes.

**Table 1:** Baseline characteristics of patients (*N* = 2488).

Demographic and anthropometric information	
Age, years, mean ± SD	65.68 ± 13.22
Sex, %	
Female	919 (36.9)
Male	1569 (63.1)
Ethnic, %	
Arab	350 (14.1)
Fars	1876 (75.4)
Others	262 (10.5)
Marital status, %	
Unmarried	45 (1.8)
Married	2443 (98.2)
Body mass index, kg/m^2^, %	
<18.5 (underweight)	165 (6.6)
18.5–24.9 (normal weight)	1193 (48.0)
≥25 (overweight)	1130 (45.4)
Waist circumference, %	
<88 (female) or 102 (male)	1675 (67.3)
≥88 (female) or 102 (male)	813 (32.7)
Place of residence	
Fasa	849 (34.1)
Other cities	929 (37.3)
Village	710 (28.5)
Drug use	
Opium, %	
Yes	686 (27.6)
No	1802 (72.4)
Classes of heart failure	
NYHA classification, %	
NYHA I	1240 (49.8)
NYHA II	544 (21.9)
NYHA III	431 (17.3)
NYHA IV	273 (11.0)
Underlying disease	
Previous chronic heart failure hospitalization, %	
No	1757 (70.6)
>30 day	601 (24.2)
<30 day	130 (5.2)
Duration of heart failure, %	
Over 6 months	712 (28.6)
Less than 6 months	1776 (71.4)
Dilated cardiomyopathy, %	
Yes	51 (2.0)
No	2437 (98.0)
RV failure, %	
Yes	4 (0.2)
No	2484 (99.8)
Previous MI, %	
Yes	1720 (69.1)
No	768 (30.9)
Atrial fibrillation/flutter, %	
Yes	220 (8.8)
No	2268 (91.2)
COPD, %	
Yes	92 (3.7)
No	2396 (96.3)
Heart valve disease, %	
Yes	282 (11.3)
No	2206 (88.7)
Previous stroke, %	
Yes	160 (6.4)
No	2328 (99.6)
Hypertension, %	
Yes	122 (4.9)
No	2366 (95.1)
Diabetes, %	
Yes	692 (27.8)
No	1796 (72.2)
LBBB, %	
Yes	295 (11.9)
No	2193 (88.1)
Treatment	
CABG, %	
Yes	319 (12.8)
No	2169 (87.2)
PCI, %	
Yes	692 (27.8)
No	1796 (72.2)
ACE Inhibitor, %	
Yes	1789 (71.9)
No	699 (28.1)
ARB, %	
Yes	506 (20.3)
No	1982 (79.7)
β‐blocker, %	
Yes	2266 (91.1)
No	222 (8.9)
Mineralocorticoid receptor antagonists (MRA), %	
Yes	1099 (44.2)
No	1389 (55.8)
Diuretics, %	
Yes	1518 (61.0)
No	970 (39.0)
Digitalis, %	
Yes	231 (9.3)
No	2257 (90.7)
Statins, %	
Yes	2434 (97.8)
No	54 (2.2)
Intravenous inotropic support type, %	
Yes	2289 (92.0)
No	199 (8.0)
Long‐acting nitrates, %	
Yes	1352 (54.3)
No	1136 (45.7)
Anticoagulants, %	
Yes	2160 (86.8)
No	328 (13.2)
ASA or antiplatelet, %	
Yes	2463 (99.0)
No	25 (1.0)
Device therapy, %	
Pacemaker	21 (0.8)
ICD	15 (0.6)
CRT‐D	11 (0.4)
No	2441 (98.1)
Lab data	
Hemoglobin, %	
<12 (female) or 13 (male)	924 (37.1)
≥12 (female) or 13 (male)	1564 (62.9)
Cholesterol, %	
<200	2223 (89.3)
≥200	265 (10.7)
Triglyceride, %	
<150	1882 (75.6)
≥150	606 (24.4)
HDL‐C, %	
<50 (female) or 40 (male)	1525 (61.3)
≥50 (female) or 40 (male)	963 (38.7)
LDL‐C, %	
<130	2251 (90.5)
≥130	237 (9.5)
Na, %	
<135	1118 (44.9)
135–145	1338 (53.8)
>145	32 (1.3)
K, %	
<3.5	301 (12.1)
3.5–5.5	2088 (83.9)
>5.5	99 (4.0)
Blood sugar, mg/dL, median (IQR)	133.57 (106.00, 191.00)
WBC, per microliter, median (IQR)	8.50 (6.60, 11.00)
Creatinine, mg/dL, median (IQR)	1.20 (1.00, 1.40)
GFR, mL/min, median (IQR)	54.17 (39.67, 70.44)
Vital signs	
Heart rate, %	
<60 (bradycardia)	224 (9.0)
60–100 (normocardia)	2073 (83.3)
>100 (tachycardia)	191 (7.7)
Systolic blood pressure, mmHg, median (IQR)	120 (110, 135)
Diastolic blood pressure, mmHg, median (IQR)	80 (70,85)
Family history	
Family history of stroke, %	
Yes	163 (6.6)
No	2325 (93.4)
Family history of heart failure, %	
Yes	192 (7.7)
No	2296 (92.3)
Family history of myocardial infarction, %	
Yes	504 (20.3)
No	1984 (79.7)
Others	
Smoking, %	
Yes	783 (31.5)
No	1705 (68.5)
Caregiver, %	
Hospital	2437 (98.0)
Clinic	51 (2.0)
ECG rhythm, %	
Sinus rhythm	2241 (90.1)
Pacemaker rhythm	28 (1.1)
Atrial fibrillation	198 (8.0)
Others	21 (0.8)
LVEF, %	
50%–70% (normal range)	11 (0.4)
40%–49% (mild dysfunction)	991 (39.8)
30%–39% (moderate dysfunction)	849 (34.1)
Less than 30% (severe dysfunction)	637 (25.6)
Length of stay in hospital (day), median (IQR)	3 (2, 5)
Outcome	
Hospital readmission, %	
Yes	366 (14.7)
No	2122 (85.3)
One‐month mortality, %	
Alive	2391 (96.1)
Death	97 (3.9)
One‐year mortality, %	
Alive	2146 (86.3)
Death	342 (13.7)

*Note*: Median (IQR).

Mean ± SD.

Number (%).

Abbreviations: ACE inhibitor, angiotensin‐converting enzyme inhibitor; ARB, angiotensin receptor blockers; ASA, acetylsalicylic acid; CABG, coronary artery bypass graft; COPD, chronic obstructive pulmonary disease; ECG, electrocardiogram; GFR, glomerular filtration rate; HDL‐C, high‐density lipoprotein cholesterol; LBBB, left bundle branch block; LDL‐C, low density lipoprotein cholesterol; LVEF, left ventricular ejection fraction; MI, myocardial infarction; NYHA classification, New York Heart Association Classification; PCI, percutaneous coronary intervention; RV failure, right ventricular failure; SD, standard deviation; WBC, white blood cell.

The mean age of the participants was 65.68 ± 13.22 years. Of the patients, 919 (36.9%) were female, and 1569 (63.1%) were male. 1876 (75.4%) people were of Fars ethnicity, while 350 (14.1%) were Arab. Only 45 (1.8%) of people were unmarried. BMI estimated that 165 (6.6%) of the patients were underweight, 1193 (48.0%) were at a normal weight, and 1130 (45.4%) were overweight.

The waist circumference (WC) of 813 (32.7%) participants was abnormal (WC ≥ 88 for females or 102 for males). According to the place of residence, 849 (34.1%) of people lived in Fasa city, 929 (37.3%) in other cities, and 710 (28.5%) in villages. In addition, 686 (2706%) persons used opium. Moreover, 1240 (49.8%), 544 (21.9%), 431 (17.3%), and 273 (11%) of the population, respectively, are classified as 1, 2, 3, and 4, according to the NYHA classification. Hospital care was given to 2438 (98%) patients, while only 50 (2%) received clinical care. In addition, 1757 (70%) of patients had never before been hospitalized for HF, compared to 130 (5.2%) who had done so less than 30 days ago and 601 (24.2%) who had done so more than 30 days ago. On the one hand, 712 (28.6%) of persons experienced HF for more than 6 months, while 1776 (71.4%) had it for less than 6 months. People based on clinical disease history, 51 (2%) had dilated cardiomyopathy, 4 (0.2%) had RV failure, 1720 (69.1%) had previous myocardial infarction (MI), 220 (8.8%) had atrial fibrillation/flutter, 92 (3.7%) had COPD, 282 (11.3%) had heart valve disease, 160 (6.4%) had a previous stroke, 295 (11.9%) had LBBB, 122 (4.9%) had hypertension, and 692 (27.8%) had diabetes.

According to the medications used, 1789 (71.9%) were using ACE inhibitors, 2266 (91.1%) were using β‐blockers, 1099 (44.2%) were using MRAs, 1518 (61%) were using diuretics, 231 (9.3%) were digitalis, 2434 (97.8%) were using statins, 1352 (54.3%) were using long‐acting nitrates, 2160 (86.8%) were using anticoagulants, and 2463 (99%) were using ASA or antiplatelet. During their hospitalization, 2289 (92%) had received intravenous inotropic. In total, 47 (1.8%) were treated with different devices, including 21 (0.8%) with a pacemaker, 15 (0.6%) with an ICD, and 11 (0.4%) with a CRT‐D. CABG surgery was done on 319 (12.8%) of the participants, whereas PCI was performed on 692 (27.8%).

According to lab data, 924 (37.1%) of persons have abnormal hemoglobin (Hb<12 [female] or 13 [male]), 265 (10.7%) have cholesterol over 200, 606 (24.4%) have triglycerides above 150, 1525 (61.3%) have abnormal HDL‐C (50 [male] or 40 [female]), and 237 (9.5%) have LDL‐C more than 130. Only 1338 (53.8%) of participants had normal sodium level. In addition, 1118 (44.9%) had hyponatremia and 32 (1.3%) had hypernatremia. Blood potassium levels were normal in 2088 (83.9%) of participants. 301 (12.1%) participants were hypokalemic, whereas 99 (4%) were hyperkalemic. The median was computed for several variables, including blood sugar (133.57 [106.00, 191.00]), WBC (8.50 [6.60, 11.00]), creatinine (1.20 [1.00, 1.40]), and GFR (54.17 [39.67, 70.44]). Patients were divided into three groups based on their heart rate: bradycardia, normocardia, and tachycardia, which contained 224 (9%), 2073 (83.3%), and 191 (7.7%) of the population, respectively. Systolic and diastolic blood pressure averages were 120 (110, 135) and 80 (70, 85), respectively. Moreover, 163 (6.6%) individuals had a family history of stroke, 192 (7.7%) people had a family history of HF, and 504 (20.3%) people had a family history of myocardial infarction. In addition, 783 (31.5%) of the population smoked. Patients were categorized into four groups based on LVEF: normal range, mild dysfunction, moderate dysfunction, and severe dysfunction, which included 11 (0.4%), 991 (39.8%), 849 (34.1%), and 637 (25.6%) of the population, respectively. The median length of hospital stay was 3 (2, 5) days. Moreover, 2241 (90.1%) of individuals had sinus rhythm, 28 (1.1%) had pacemaker rhythm, and 198 (8%) had atrial fibrillation, according to ECG rhythm.

### 4.2 Comparison of the performance of the machine algorithms

#### 4.2.1 Hospital readmission

The greatest AUC was associated with GNB (0.75), followed by CAT and LDA (0.74), LR and RF (0.73), and XGB (0.71). DT presented the least AUC (0.54). The AUC of the remaining models was 0.67, 0.63, and 0.59 for GBM, SVM, and KNN, respectively. CAT also had the highest F1 score with a value of 0.91. Table 2 contains all of the remaining information.

**Table 2:** Performance of the machine learning algorithms based on hospital readmission.

Algorithm	Accuracy	Sensitivity	Specificity	F1 score	AUC
LR	0.77 (0.72–0.82)	0.81 (0.77–0.85)	0.52 (0.45–0.59)	0.86 (0.82–0.9)	0.73 (0.68–0.79)
DT	0.69 (0.63–0.75)	0.75 (0.7–0.8)	0.36 (0.29–0.43)	0.81 (0.77–0.85)	0.54 (0.47–0.61)
SVM	0.77 (0.72–0.82)	0.84 (0.8–0.88)	0.36 (0.29–0.43)	0.86 (0.82–0.9)	0.63 (0.57–0.7)
RF	0.82 (0.78–0.86)	0.92 (0.9–0.94)	0.27 (0.2–0.34)	0.9 (0.87–0.93)	0.73 (0.67–0.78)
GNB	0.69 (0.63–0.75)	0.7 (0.64–0.76)	0.64 (0.58–0.7)	0.79 (0.74–0.84)	0.75 (0.7–0.8)
LDA	0.78 (0.73–0.83)	0.82 (0.78–0.86)	0.55 (0.48–0.62)	0.86 (0.82–0.9)	0.74 (0.69–0.8)
KNN	0.69 (0.63–0.75)	0.74 (0.69–0.79)	0.44 (0.37–0.51)	0.81 (0.77–0.85)	0.59 (0.52–0.66)
GBM	0.84 (0.8–0.88)	0.94 (0.92–0.96)	0.26 (0.19–0.33)	0.88 (0.85–0.91)	0.67 (0.61–0.73)
XGB	0.83 (0.79–0.87)	0.93 (0.91–0.95)	0.27 (0.2–0.34)	0.9 (0.87–0.93)	0.71 (0.66–0.77)
CAT	0.79 (0.74–0.84)	0.86 (0.82–0.9)	0.4 (0.33–0.47)	0.91 (0.88–0.94)	0.74 (0.68–0.79)

Abbreviations: AUC, area under the curve; CAT, Cat boost; DT, decision tree; GBM, gradient boosting machine; GNB, Gaussian Naive Bayes; KNN, K‐nearest neighbors; LDA, linear discriminant analysis; LR, logistic regression; RF, random forest; SVM, support vector machine; XGB, extreme gradient boosting.

#### 4.2.2 One‐month mortality

Ten models were used to predict 1‐month mortality; the best AUC was associated with RF (0.62), followed by CAT and GNB (0.61), LR, GBM, LDA (0.60), and XGB (0.56). KNN had the least AUC (0.51). The remaining models were 0.55, and 0.52 for DT and SVM, respectively. Furthermore, CAT has the highest F1 score with a value of 0.13. Table 3 contains all of the remaining details.

**Table 3:** Performance of the machine learning algorithms based on 1‐month mortality.

Algorithm	Accuracy	Sensitivity	Specificity	F1 score	AUC
LR	0.85 (0.74–0.96)	0.26 (0.17–0.35)	0.87 (0.77–0.97)	0.11 (0.06–0.16)	0.6 (0.46–0.73)
DT	0.87 (0.77–0.97)	0.21 (0.13–0.29)	0.89 (0.79–0.99)	0.11 (0.06–0.16)	0.55 (0.42–0.69)
SVM	0.9 (0.81–0.99)	0.05 (0.02–0.08)	0.93 (0.85–1)	0.04 (0.02–0.06)	0.52 (0.39–0.66)
RF	0.93 (0.85–1)	0.0 (0.0–0.0)	0.97 (0.92–1)	0.0 (0.0–0.0)	0.62 (0.49–0.76)
GNB	0.75 (0.62–0.88)	0.42 (0.3–0.54)	0.76 (0.63–0.89)	0.11 (0.06–0.16)	0.61 (0.47–0.75)
LDA	0.9 (0.81–0.99)	0.11 (0.06–0.16)	0.93 (0.85–1)	0.07 (0.04–0.1)	0.6 (0.46–0.73)
KNN	0.83 (0.72–0.94)	0.16 (0.09–0.23)	0.86 (0.75–0.97)	0.07 (0.04–0.1)	0.51 (0.38–0.64)
GBM	0.94 (0.87–1)	0.0 (0.0–0.0)	0.97 (0.92–1)	0.0 (0.0–0.0)	0.6 (0.46–0.73)
XGB	0.93 (0.85–1)	0.0 (0.0–0.0)	0.97 (0.92–1)	0.0 (0.0–0.0)	0.56 (0.43–0.7)
CAT	0.81 (0.69–0.93)	0.37 (0.25–0.49)	0.83 (0.72–0.94)	0.13 (0.07–0.19)	0.61 (0.47–0.75)

Abbreviations: AUC, area under the curve; CAT, Cat boost; DT, decision tree; GBM, gradient boosting machine; GNB, Gaussian Naive Bayes; KNN, K‐nearest neighbors; LDA, linear discriminant analysis; LR, logistic regression; RF, random forest; SVM, support vector machine; XGB, extreme gradient boosting.

#### 4.2.3 One‐year mortality

To predict 1‐year mortality, 10 models were utilized. The model with the highest AUC was GNB (0.59), followed by CAT, LR, RF, LDA, KNN (0.58), GBM, XGB, SVM (0.56), and DT (0.51). RF, GBM, XGB, and CAT also had the highest F1 score (0.88). Table 4 contains all of the remaining information.

**Table 4:** Performance of the machine learning algorithms based on 1‐year mortality.

Algorithm	Accuracy	Sensitivity	Specificity	F1 score	AUC
LR	0.58 (0.51–0.65)	0.6 (0.53–0.67)	0.44 (0.37–0.51)	0.71 (0.65–0.77)	0.58 (0.51–0.65)
DT	0.72 (0.66–0.78)	0.8 (0.75–0.85)	0.25 (0.18–0.32)	0.83 (0.79–0.87)	0.51 (0.43–0.57)
SVM	0.72 (0.66–0.78)	0.8 (0.75–0.85)	0.29 (0.22–0.36)	0.83 (0.79–0.87)	0.56 (0.49–0.63)
RF	0.8 (0.75–0.85)	0.92 (0.9–0.94)	0.11 (0.06–0.16)	0.88 (0.85–0.91)	0.58 (0.51–0.65)
GNB	0.54 (0.47–0.61)	0.53 (0.46–0.6)	0.56 (0.49–0.63)	0.66 (0.6–0.72)	0.59 (0.52–0.65)
LDA	0.57 (0.5–0.64)	0.59 (0.52–0.66)	0.44 (0.37–0.51)	0.7 (0.64–0.76)	0.58 (0.51–0.65)
KNN	0.66 (0.6–0.72)	0.69 (0.63–0.75)	0.47 (0.4–0.54)	0.77 (0.72–0.82)	0.58 (0.51–0.65)
GBM	0.8 (0.75–0.85)	0.92 (0.9–0.94)	0.08 (0.04–0.12)	0.88 (0.85–0.91)	0.56 (0.5–0.63)
XGB	0.79 (0.74–0.84)	0.91 (0.88–0.94)	0.1 (0.05–0.15)	0.88 (0.85–0.91)	0.56 (0.49–0.63)
CAT	0.72 (0.66–0.78)	0.8 (0.75–0.85)	0.26 (0.19–0.33)	0.88 (0.85–0.91)	0.58 (0.51–0.65)

Abbreviations: AUC, area under the curve; CAT, Cat boost; DT, decision tree; GBM, gradient boosting machine; GNB, Gaussian Naive Bayes; KNN, K‐nearest neighbors; LDA, linear discriminant analysis; LR, logistic regression; RF, random forest; SVM, support vector machine; XGB, extreme gradient boosting.

In addition, in Supporting Information S1: Figure 2, the AUC of all models is represented visually, separated by three outcomes.

### 4.3 Feature importance

Because the CAT model performed best for practically all three of our outcomes, it was utilized to identify and rank the most important factors. Figure 1 displays significant predictors for each outcome. The length of stay in the hospital was the most important predictor of hospital readmission, followed by medical history of CABG, blood sugar, systolic blood pressure, and intravenous inotropic support type. Hemoglobin level was the most important predictor of 1‐month mortality, followed by age, prior HF hospitalization, Body mass index, gender, and sodium level. Family history of MI was the most significant predictor of 1‐year mortality, followed by sodium level, body mass index, length of stay in the hospital, systolic blood pressure, and diastolic blood pressure.

## 5 DISCUSSION

This retrospective cohort study^23^ settled and validated 10 ML models to predict mortality and readmission of patients with HF. The current study supports using the CAT algorithm for risk evaluation in the medical care of HF patients. The CAT algorithm outperformed LR, DT, GNB, RF, LAD, SVM, XGB, KNN, and GBM.

This study recognized some essential variables related to rehospitalization and mortality of patients with HF. In this study, length of stay in the hospital, medical history of CABG, blood sugar, systolic blood pressure, and intravenous inotropic support type were known as the most significant predictor variables related to readmission. The hemoglobin level was the most important predictor of 1‐month mortality, followed by age, prior HF hospitalization, Body mass index, gender, and sodium level. Family history of MI was the most critical predictor of 1‐year mortality.

Several studies have predicted readmission and mortality among HF patients using different ML models. Although, it is hard to compare the outcomes of these studies because each study evaluated various features of HF patients.^35^

Li et al. and Sun et al. showed that CAT had been used to predict the mortality of patients and may help the physician in decision‐making.^24^, ^36^ The same technique can be used in any cohort research, allowing the algorithm to be revalidated over time to reflect an active population. Finally, predictive ML aims to recognize vulnerable HF patients and take action in the postdischarge period to reduce the future risk of HF. There was no evidence that CAT could be used to predict readmission. For example, Sun et al. developed and validated seven algorithms to predict the mortality of HF patients with hypoxic hepatitis (HH). In these algorithms, the authors used the Kaplan–Meier and multivariate Cox analysis to determine the effect of HH on the mortality of HF patients. Internal and external validation suggested that the CAT algorithm had a higher ability than the other algorithms (internal validation: AUC, 0.832; 95% CI, 0.819–0.845; external validation: AUC, 0.757 95% CI, 0.739–0.776).^36^ In another study, Li et al. validated 11 ML algorithms to predict mortality in mechanically ventilated patients with HF; that CAT algorithm was validated using an external validation and showed the best performance (AUC = 0.806).^24^ The CAT algorithm works by building a set of decision trees, where each new tree is trained to correct previous errors. This is done sequentially, meaning that each tree is built using information from previous trees. This iterative process continues until a certain stopping criterion such as maximum number of trees or minimum error rate is met.^37^ Perhaps one of the reasons why CAT performed better in our study and in some previous studies was this.

But Awan et al. showed that multilayer perceptron (MLP) with the highest AUC (0.62), AUPRC (0.46), sensitivity (48%), and specificity (70%) had the best performance in predicting death or readmission of HF patients.^38^

Angraal et al. assessed five ML algorithms that RF had the best performance in predicting HF hospitalization and mortality, with a mean C‐statistic of 0.72 for predicting mortality and 0.76 for HF hospitalization. BUN levels, BMI, and Kansas City Cardiomyopathy Questionnaire (KCCQ) subscale scores were powerfully associated with mortality. In contrast, Hb level, BUN, time since previous HF hospitalization, and KCCQ scores were the most important predictors of hospitalization.^23^

Peng et al. validated four models to predict the mortality of ICU patients with HF combined with hypertension. The neural networks (NN) model had the best predictive performance with an AUC of 0.764 and 0.674 in the test and external validation cohort, respectively.^25^ Landicho et al. and Artetxe et al. reported that the SVM had better performance in predicting the readmission of HF patients than other algorithms.^39^, ^40^

In our study, the most important predictors for readmission, 1‐month mortality, and 1‐year mortality were, respectively, length of stay in the hospital, hemoglobin level, and family history of MI. In the previous study,^41^ there was a relationship between the length of stay in the hospital and readmission. Still, the previous study,^41^ unlike our study, showed a negative relationship between the length of stay and the possibility of readmission, especially in the case of a heart attack. Extending the length of stay for some patients may be a means to improve the quality of care by reducing readmissions during the 30‐day postdischarge period, they stated in their study. The difference was seen, maybe because, in our study, the length of stay in the hospital indicates a worse condition of the disease, which leads to readmission for HF patients.

Regarding 1‐month mortality, as seen in our study, the importance of hemoglobin level was confirmed. In a previous study,^42^ it was also stated that patients with anemia were more exposed to in‐hospital complications such as HF, frequent ischemia, reinfarction, cardiogenic shock, stroke, and major bleeding. Hemoglobin level has a significant effect on the prognosis of HF patients, but other factors such as the age of the patients and the type of HF should also be considered.^43^ Also, this study states that anemia or in‐hospital mortality is associated with 1‐month and 1‐year mortality. Therefore, anemia and other risk scores should be considered in the initial risk assessment.

Previous studies showed that age was the main predictor of mortality and readmission of HF patients by ML models.^25^, ^35^, ^36^, ^44^ When HF gets worse, particularly in old age, it can cause severe ischemia, respiratory failure, and death.^25^ It has been emphasized in various studies^45^, ^46^, ^47^, ^48^, ^49^ that older age has been consistently associated with worse outcomes. This issue can be critical, especially considering the aging of the population and the effect of age on HF. Of course, our study did not deal with the nonlinear relationship of age, which can be a topic investigated in other studies.

Also, our study is similar to the previous study^50^ and shows the association between a family history of MI and HF mortality. In the previous study, the family history of MI is an independent risk factor for coronary heart disease (CHD) mortality, which differs in terms of the effect based on the gender of the index person and the type of family relationship. The life‐course socioeconomic position has little impact on the association between family history and CHD, suggesting that this factor does not confound this effect. This issue may indicate the conditions of stable family eating habits, the same environmental conditions, or genetics that can affect the mortality of HF, which can be specifically investigated in other studies.

As a retrospective analysis, this study had limitations. First, our algorithm was made from a center data set that may not be suitable for another population. But, our algorithm performed well in the internal data set because all cardiac patients from Fasa and the peripheral village came to this center. Second, our models performance, dependent on the data sets accuracy, was collected from patients, so we used trained Employees to collect data. Third, the data set did not contain patients' psychosocial information, which might improve the performance of ML models. Despite these limitations, ML models have a primary role in preventing rehospitalization, reducing mortality, improving patients' quality of life, and decreasing health costs.

## 6 CONCLUSION

This study recognized some essential variables related to rehospitalization and mortality of patients with HF. In this study, length of stay in the hospital, hemoglobin level, and family history of MI were known as the most significant predictor variables related to readmission, 1‐month mortality, and 1‐year mortality. Health policymakers and managers should pay attention to these features to reduce mortality and readmission of HF patients by improving the quality of life, paying attention to the elderly, and providing free health care services. Doctors and clinical staff can identify HF patients as soon as possible and do the necessary procedures to prevent disease development. In fact, predictive models based on machine learning can help doctors identify HF patients at high risk of readmission or death. This information can help doctors take more preventive measures, such as adjusting medications or providing special care services.

## AUTHOR CONTRIBUTIONS

All authors made significant contributions to the design of the study, gathering and analyzing the data, and drafting the manuscript. Marzieh Ketabi, Aref Andishgar, Mohebat Vali and Reza Tabrizi contributed to drafting the article or revising it. Marzieh Ketabi, Zhila Fereidouni, Maryam Mojarrad Sani, Ashkan Abdollahi and Abdulhakim Alkamel approved the revised version to be submitted. All authors have contributed to the work, reviewed and approved the final version of the manuscript.

## CONFLICT OF INTEREST STATEMENT

The authors declare no conflict of interest.

## Supporting information

Supporting information.

## Data Availability

If someone wants to request the data from this study, corresponding authors should be contacted.
