# Supplementary material for: Predicting the risk of mortality and rehospitalization in heart failure patients: A retrospective cohort study by machine learning approach
Source: Clin Cardiol. 2024 Feb 25;47(2):e24239. doi: 10.1002/clc.24239 (PMC10894620; doi:10.1002/clc.24239)
Supplement: Supplementary file 1 — Supporting information. [file CLC-47-e24239-s001.docx]

**Predicting the Risk of Mortality and Hospitalization in Heart Failure Using Machine Learning Algorithms**

Supplementary Appendix

| **Content** | **Page Number** |
| --- | --- |
| **Supplementary Table 1**. The International Classification of Diseases, Tenth Revision (ICD-10) coding | 2 |
| **Supplementary Table 2**. Characteristics of patients based on hospital readmission | 3 |
| **Supplementary Table 3**. Characteristics of patients based on one-month mortality | 7 |
| **Supplementary Table 4**. Characteristics of patients based on one-year mortality | 10 |
| **Supplementary Figure 1**. All stages for implementing machine learning algorithms | 14 |
| **Supplementary Figure 2**. The receiver operating characteristic curve among the ten models for prediction of hospital readmission, one-month mortality and one-year mortality | 15 |

| **Supplementary Table 1.** the International Classification of Diseases, Tenth Revision (ICD-10) coding | |
| --- | --- |
| **ICD-10 codes** | **Long title** |
| I509 | Heart failure, unspecified |
| I110 | Hypertensive heart disease with heart failure |
| I132 | Hypertensive heart and chronic kidney disease with heart failure and with stage 5 chronic kidney disease, or end stage renal disease |
| I5020 | Unspecified systolic (congestive) heart failure |
| I5021 | Acute systolic (congestive) heart failure |
| I5022 | Chronic systolic (congestive) heart failure |
| I5023 | Acute on chronic systolic (congestive) heart failure |
| I5030 | Unspecified diastolic (congestive) heart failure |
| I5031 | Acute diastolic (congestive) heart failure |
| I5032 | Chronic diastolic (congestive) heart failure |
| I5033 | Acute on chronic diastolic (congestive) heart failure |
| I5040 | Unspecified combined systolic (congestive) and diastolic (congestive) heart failure |
| I5041 | Acute combined systolic (congestive) and diastolic (congestive) heart failure |
| I5042 | Chronic combined systolic (congestive) and diastolic (congestive) heart failure |
| I5043 | Acute on chronic combined systolic (congestive) and diastolic (congestive) heart failure |

| Supplementary Table 2: characteristics of patients based on hospital readmission (N=2488) | | | | |
| --- | --- | --- | --- | --- |
|  |  | **Hospital readmission** | | **P.value** |
|  |  | **Yes** | **NO** |  |
| Demographic and anthropometric information | | | | |
| Age, years, Mean±SD |  | 68.02±12.18 | 65.28±13.34 | 0.064^a^ |
| SEX, % | **Female** | 140(15.2) | 779(84.8) | 0.573^b^ |
|  | **Male** | 226(14.4) | 1343(85.6) |  |
| Ethnic, % | **Arab** | 83(23.7) | 267(76.3) | **≤0.001^b^** |
|  | **Fars** | 231(12.3) | 1645(87.7) |  |
|  | **Other** | 52(19.8) | 210(80.2) |  |
| Marital status, % | **Single** | 4(8.9) | 41(91.1) | 0.266^b^ |
|  | **Married** | 362(14.8) | 2081(85.2) |  |
| Body-mass index, kg/m², % | **<18.5** | 25(15.2) | 140(84.8) | 0.890^b^ |
|  | **18.5 to 24.9** | 179(15.0) | 1014(85.0) |  |
|  | **>=24.9** | 162(14.3) | 968(85.7) |  |
| Waist Circumference categorize, % | **<88 or 102** | 224(13.4) | 1451(86.6) | **0.007^b^** |
|  | **>=88 or 102** | 142(17.5) | 671(82.5) |  |
| Place of residence | **Fasa** | 155(18.3) | 694(81.7) | **≤0.001^b^** |
|  | **Other city** | 47(5.1) | 882(94.9) |  |
|  | **Village** | 164(23.1) | 546(76.9) |  |
| Drug Use |  |  |  |  |
| Smoking, % | **Yes** | 113(14.4) | 670(85.6) | 0.790^b^ |
|  | **No** | 253(14.8) | 1452(85.2) |  |
| Opium, % | **Yes** | 280(15.5) | 1522(84.5) | 0.059^b^ |
|  | **No** | 86(12.5) | 600(87.5) |  |
| Classes of Heart Failure |  |  |  |  |
| NYHA classification, % | **NYHA I** | 107(8.6) | 1133(91.4) | **≤0.001^b^** |
|  | **NYHA II** | 104(19.1) | 440(80.9) |  |
|  | **NYHA III** | 101(23.4) | 330(76.6) |  |
|  | **NYHA IV** | 54(19.8) | 219(80.2) |  |
| Underlying disease |  |  |  |  |
| Caregiver, % | **Hospital** | 360(14.8) | 2077(85.2) | 0.548^b^ |
|  | **Clinic** | 6(11.8) | 45(88.2) |  |
| Previous chronic heart failure hospitalization, % | **NO** | 148(8.4) | 1609(91.6) | **≤0.001^b^** |
|  | **>30 day** | 183(30.4) | 418(69.6) |  |
|  | **<30 day** | 35(26.9) | 95(73.1) |  |
| Duration of heart failure, % | **Over 6 months** | 218(30.6) | 494(69.4) | **≤0.001^b^** |
|  | **Less than 6 months** | 148(8.3) | 1628(91.7) |  |
| Dilated cardiomyopathy, % | **Yes** | 19(37.3) | 32(62.7) | **≤0.001^b^** |
|  | **No** | 347(14.2) | 2090(85.8) |  |
| RV failure, % | **Yes** | 0(0.0) | 4(100.0) | 0.406^b^ |
|  | **No** | 366(14.7) | 2118(85.3) |  |
| Previous MI, % | **Yes** | 194(11.3) | 1526(88.7) | **≤0.001^b^** |
|  | **No** | 172(22.4) | 596(77.6) |  |
| Atrial fibrillation/flutter, % | **Yes** | 57(25.9) | 163(74.1) | **≤0.001^b^** |
|  | **No** | 309(13.6) | 1959(86.4) |  |
| COPD, % | **Yes** | 33(35.9) | 59(64.1) | **≤0.001^b^** |
|  | **No** | 333(13.9) | 2063(86.1) |  |
| Heart valve disease, % | **Yes** | 63(22.3) | 219(77.7) | **≤0.001^b^** |
|  | **No** | 303(13.7) | 1903(86.3) |  |
| Previous stroke, % | **Yes** | 32(20.0) | 128(80.0) | 0.051 |
|  | **No** | 334(14.3) | 1994(85.7) |  |
| Hypertension, % | **Yes** | 34(27.9) | 88(72.1) | **≤0.001^b^** |
|  | **No** | 332(14.0) | 2034(86.0) |  |
| Diabetes, % | **Yes** | 125(18.1) | 567(81.9) | **0.003^b^** |
|  | **No** | 241(13.4) | 1555(86.6) |  |
| Treatment |  |  |  |  |
| CABG, % | **Yes** | 96(30.1) | 223(69.9) | **≤0.001^b^** |
|  | **No** | 270(12.4) | 1899(87.6) |  |
| PCI, % | **Yes** | 114(16.5) | 578(83.5) | 0.123^b^ |
|  | **No** | 252(14.0) | 1544(86.0) |  |
| ECG rhythm, % | **Sinus rhythm** | 310(13.8) | 1931(86.2) | **≤0.001^b^** |
|  | **Pacemaker rhythm** | 10(35.7) | 18(64.3) |  |
|  | **Atrial fibrillation** | 4(19.0) | 17(81.0) |  |
|  | **Other** | 42(21.2) | 156(78.8) |  |
| LBBB, % | **Yes** | 73(24.7) | 222(75.3) | **≤0.001^b^** |
|  | **No** | 293(13.4) | 1900(86.6) |  |
| ACE Inhibitor, % | **Yes** | 241(13.5) | 1548(86.5) | **0.005^b^** |
|  | **No** | 125(17.9) | 574(82.1) |  |
| ARB, % | **Yes** | 102(20.2) | 404(79.8) | **≤0.001^b^** |
|  | **No** | 264(13.3) | 1718(86.7) |  |
| Beta blocker, % | **Yes** | 319(14.1) | 1947(85.9) | **0.004^b^** |
|  | **No** | 47(21.2) | 175(78.8) |  |
| Mineralocorticoid Receptor Antagonists(MRA) , % | **Yes** | 219(19.9) | 880(80.1) | **≤0.001^b^** |
|  | **No** | 147(10.6) | 1242(89.4) |  |
| Diuretics, % | **Yes** | 213(22.0) | 757(78.0) | **≤0.001^b^** |
|  | **No** | 153(10.1) | 1365(89.9) |  |
| Digitalis, % | **Yes** | 85(36.8) | 146(63.2) | **≤0.001^b^** |
|  | **No** | 281(12.5) | 1976(87.5) |  |
| Statins, % | **Yes** | 358(14.7) | 2076(85.3) | 0.983^b^ |
|  | **No** | 8(14.8) | 46(85.2) |  |
| i.v. inotropic support type, % | **Yes** | 344(15.0) | 1945(85.0) | 0.129 |
|  | **No** | 22(11.1) | 177(88.9) |  |
| Long acting nitrates, % | **Yes** | 253(18.7) | 1099(81.3) | **≤0.001^b^** |
|  | **No** | 113(9.9) | 1023(90.1) |  |
| Anticoagulants, % | **Yes** | 354(16.4) | 1806(83.6) | **≤0.001^b^** |
|  | **No** | 12(3.7) | 316(96.3) |  |
| ASA or Antiplatelet, % | **Yes** | 365(14.8) | 2098(85.2) | 0.129^b^ |
|  | **No** | 1(4.0) | 24(96.0) |  |
| Device Therapy, % | **Pace Maker** | 7(33.3) | 14(66.7) | **0.017^b^** |
|  | **ICD** | 5(33.3) | 10(66.7) |  |
|  | **CRT-D** | 2(18.2) | 9(81.8) |  |
|  | **No** | 352(14.4) | 2089(85.6) |  |
| Lab data |  |  |  |  |
| Hemoglobin, % | **<12 or 13** | 154(16.7) | 770(83.3) | **0.034^b^** |
|  | **>= 12 or 13** | 212(13.6) | 1352(86.4) |  |
| Cholesterol, % | **<200** | 326(14.7) | 1897(85.3) | 0.852^b^ |
|  | **>=200** | 40(15.1) | 225(84.9) |  |
| Triglyceride, % | **<150** | 297(15.8) | 1585(84.2) | **0.008^b^** |
|  | **>=150** | 69(11.4) | 537(88.6) |  |
| HDL, % | **<50 or <40** | 233(15.3) | 1292(84.7) | 0.314^b^ |
|  | **>=50 or >=40** | 133(13.8) | 830(86.2) |  |
| LDL, % | **<130** | 325(14.4) | 1926(85.6) | 0.237^b^ |
|  | **>=130** | 41(17.3) | 196(82.7) |  |
| Na, % | **<135** | 178(15.9) | 940(84.1) | 0.070^b^ |
|  | **135 to 145** | 187(14.0) | 1151(86.0) |  |
|  | **>=145** | 1(3.1) | 31(96.9) |  |
| K, % | **<3.5** | 33(11.0) | 268(89.0) | 0.142^b^ |
|  | **3.5 to 5.5** | 317(15.2) | 1771(84.8) |  |
|  | **>=5.5** | 16(16.2) | 83(83.8) |  |
| LVEF, % | **50% to 70% normal range** | 0(0.0) | 11(100.0) | **≤0.001^b^** |
|  | **40% to 49 mild dysfunction** | 99(10.0) | 892(90.0) |  |
|  | **30% to 39% moderate dysfunction** | 125(14.7) | 724(85.3) |  |
|  | **Less than 30% severe dysfunction** | 142(22.3) | 495(77.7) |  |
| Blood Sugar, mg/dl, Median(INQ) | | 124.00(103.00, 182.25) | 135.00(107.00, 191.55) | **0.050^c^** |
| WBC, per microliter, Median(INQ) | | 7.90(6.20,10.20) | 8.60(6.70, 11.10) | **≤0.001^c^** |
| Creatinine, mg/dl, Median(INQ) | | 1.20(1.00,1.50) | 1.20(1.00,1.40) | **0.012^c^** |
| GFR, ml/min, Median(INQ) | | 49.63(36.83,64.39) | 54.86(40.28,71.43) | **≤0.001^c^** |
| Vital Signs |  |  |  |  |
| HR, % | **<60** | 29(12.9) | 195(87.1) | 0.564^c^ |
|  | **60 to100** | 312(15.1) | 1761(84.9) |  |
|  | **>100** | 25(13.1) | 166(86.9) |  |
| Systolic, mmHg, Median(INQ) | | 120.00(110.00,140.00) | 120.00(110.00, 135.00) | 0.766^c^ |
| Diastolic, mmHg, Median(INQ) | | 80.00(70.00,85.25) | 80.00(70.00,85.00) | 0.802^c^ |
| Length of stay in hospital, Median(INQ) | | 4.00(3.00,6.00) | 3.00(2.00,5.00) | **0.001^c^** |
| Family History |  |  |  |  |
| Family History stroke, % | **Yes** | 342(14.7) | 1983(85.3) | 0.996^b^ |
|  | **No** | 24(14.7) | 139(85.3) |  |
| Family History heart failure, % | **Yes** | 310(13.5) | 1986(86.5) | **≤0.001^b^** |
|  | **No** | 56(29.2) | 136(70.8) |  |
| Family History myocardial infarction, % | **Yes** | 310(15.6) | 1674(84.4) | **0.011^b^** |
|  | **No** | 56(11.1) | 448(88.9) |  |
| Median(INQ)  Mean±SD  Number (%)  a: Independent Samples Test  b: Chi-Square  c: Mann-Whitney Test | | |  |  |
| NYHA classification: New York Heart Association Classification, RV failure: Right Ventricular Failure, MI: Myocardial Infarction, COPD: Chronic obstructive Pulmonary Disease, LBBB: Left Bundle Branch Block, CABG: Coronary Artery Bypass Graft, PCI: Percutaneous Coronary Intervention, ACE Inhibitor: Angiotensin-Converting Enzyme Inhibitor, ARB: Angiotensin Receptor Blockers, ASA: Acetylsalicylic Acid, HDL-C: High Density Lipoprotein Cholesterol, LDL-C: Low Density Lipoprotein Cholesterol, WBC: White Blood Cell, GFR: Glomerular Filtration Rate, ECG: Electrocardiogram, LVEF: Left Ventricular Ejection Fraction | | | | |

| Supplementary Table 3: characteristics of patients based on one-month mortality (N=2488) | | | | |
| --- | --- | --- | --- | --- |
|  |  | **Status (1 month)** | | **P.value** |
|  |  | **Alive** | **Death** |  |
| Demographic and anthropometric information | | | | |
| Age, years, Mean±SD |  | 65.34±13.17 | 74.11±11.42 | **≤0.001^a^** |
| SEX, % | **Female** | 878(95.5) | 41(4.5) | 0.267^b^ |
|  | **Male** | 1513(96.4) | 56(3.6) |  |
| Ethnic, % | **Arab** | 339(96.9) | 11(3.1) | 0.636^b^ |
|  | **Fars** | 1799(95.9) | 77(4.1) |  |
|  | **Other** | 253(96.6) | 9(3.4) |  |
| Marital status, % | **Single** | 42(93.3) | 3(6.7) | 0.333^b^ |
|  | **Married** | 2349(96.2) | 94(3.8) |  |
| Body-mass index, kg/m², % | **<18.5** | 153(92.7) | 12(7.3) | **0.035^b^** |
|  | **18.5 to 24.9** | 1144(95.9) | 49(4.1) |  |
|  | **>=24.9** | 1094(96.8) | 36(3.2) |  |
| Waist Circumference categorize, % | **<88 or 102** | 1607(95.6) | 68(4.1) | 0.552^b^ |
|  | **>=88 or 102** | 784(96.4) | 29(3.6) |  |
| Place of residence | **Fasa** | 813(95.8) | 36(4.2) | 0.754^b^ |
|  | **Other city** | 896(96.4) | 33(3.6) |  |
|  | **Village** | 682(96.1) | 28(3.9) |  |
| Drug Use |  |  |  |  |
| Smoking, % | **Yes** | 756(96.9) | 24(3.1) | 0.145^b^ |
|  | **No** | 1632(95.7) | 73(4.3) |  |
| Opium, % | **Yes** | 664(96.8) | 22(3.2) | 0.271^b^ |
|  | **No** | 1727(95.8) | 75(4.2) |  |
| Classes of Heart Failure |  |  |  |  |
| NYHA classification, % | **NYHA I** | 1202(96.9) | 38(3.1) | 0.119^b^ |
|  | **NYHA II** | 521(95.8) | 23(4.2) |  |
|  | **NYHA III** | 407(94.4) | 24(5.6) |  |
|  | **NYHA IV** | 261(95.6) | 12(4.4) |  |
| Underlying disease |  |  |  |  |
| Caregiver, % | **Hospital** | 2342(96.1) | 95(3.9) | 0.993^b^ |
|  | **Clinic** | 49(96.1) | 2(3.9) |  |
| Previous chronic heart failure hospitalization, % | **NO** | 1697(96.6) | 60(3.4) | 0.151^b^ |
|  | **>30 day** | 571(95.0) | 30(5.0) |  |
|  | **<30 day** | 123(94.6) | 7(5.4) |  |
| Duration of heart failure, % | **Over 6 months** | 675(94.8) | 37(5.2) | **0.034^b^** |
|  | **Less than 6 months** | 1716(96.6) | 60(3.4) |  |
| Dilated cardiomyopathy, % | **Yes** | 51(100.0) | 0(0.0) | 0.146^b^ |
|  | **No** | 2340(96.0) | 97(4.0) |  |
| RV failure, % | **Yes** | 4(100.0) | 0(0.0) | 0.687^b^ |
|  | **No** | 2387(96.1) | 97(3.9) |  |
| Previous MI, % | **Yes** | 1656(96.3) | 64(3.7) | 0.493^b^ |
|  | **No** | 735(95.7) | 33(4.3) |  |
| Atrial fibrillation/flutter, % | **Yes** | 206(93.6) | 14(6.4) | **0.048^b^** |
|  | **No** | 2185(96.3) | 83(3.7) |  |
| COPD, % | **Yes** | 85(92.4) | 7(7.6) | 0.061^b^ |
|  | **No** | 2306(96.2) | 90(3.8) |  |
| Heart valve disease, % | **Yes** | 270(95.7) | 12(4.3) | 0.742^b^ |
|  | **No** | 2121(96.1) | 85(3.9) |  |
| Previous stroke, % | **Yes** | 154(96.3) | 6(3.8) | 0.920^b^ |
|  | **No** | 2237(96.1) | 91(3.9) |  |
| Hypertension, % | **Yes** | 117(95.9) | 5(4.1) | 0.907^b^ |
|  | **No** | 2274(96.1) | 92(3.9) |  |
| Diabetes, % | **Yes** | 665(96.1) | 27(3.9) | 0.996^b^ |
|  | **No** | 1726(96.1) | 70(3.9) |  |
| Treatment |  |  |  |  |
| CABG, % | **Yes** | 301(94.4) | 18(5.6) | 0.085^b^ |
|  | **No** | 2090(96.4) | 79(3.6) |  |
| PCI, % | **Yes** | 672(97.1) | 20(2.9) | 0.107^b^ |
|  | **No** | 1719(95.7) | 77(4.3) |  |
| ECG rhythm, % | **Sinus rhythm** | 2159(96.3) | 82(3.7) | 0.151^b^ |
|  | **Pacemaker rhythm** | 25(89.3) | 3(10.7) |  |
|  | **Atrial fibrillation** | 20(95.2) | 1(4.8) |  |
|  | **Other** | 187(94.4) | 11(5.6) |  |
| LBBB, % | **Yes** | 284(96.3) | 11(3.7) | 0.872^b^ |
|  | **No** | 2107(96.1) | 86(3.9) |  |
| ACE Inhibitor, % | **Yes** | 1730(96.7) | 59(3.3) | **0.013^b^** |
|  | **No** | 661(94.6) | 38(5.4) |  |
| ARB, % | **Yes** | 483(95.5) | 23(4.5) | 0.400^b^ |
|  | **No** | 1908(96.3) | 74(3.7) |  |
| Beta blocker, % | **Yes** | 2184(96.4) | 82(3.6) | **0.021^b^** |
|  | **No** | 207(93.2) | 15(6.8) |  |
| Mineralocorticoid Receptor Antagonists(MRA) , % | **Yes** | 1049(95.5) | 50(4.5) | 0.136^b^ |
|  | **No** | 1342(96.6) | 47(3.4) |  |
| Diuretics, % | **Yes** | 916(94.4) | 54(5.6) | **0.001^b^** |
|  | **No** | 1475(97.2) | 43(2.8) |  |
| Digitalis, % | **Yes** | 220(95.2) | 11(4.8) | 0.477^b^ |
|  | **No** | 2171(96.2) | 86(3.8) |  |
| Statins, % | **Yes** | 2338(96.1) | 96(3.9) | 0.432^b^ |
|  | **No** | 53(98.1) | 1(1.9) |  |
| i.v. inotropic support type, % | **Yes** | 184(92.5) | 15(7.5) | **0.006^b^** |
|  | **No** | 2207(96.4) | 82(3.6) |  |
| Long acting nitrates, % | **Yes** | 1303(96.4) | 49(3.6) | 0.440^b^ |
|  | **No** | 1088(95.8) | 48(4.2) |  |
| Anticoagulants, % | **Yes** | 2070(95.8) | 90(4.2) | 0.076^b^ |
|  | **No** | 321(97.9) | 7(2.1) |  |
| ASA or Antiplatelet, % | **Yes** | 2366(96.1) | 97(3.9) | 0.311^b^ |
|  | **No** | 25(100.0) | 0(0.0) |  |
| Device Therapy, % | **Pace Maker** | 19(90.5) | 2(9.5) | **≤0.001^b^** |
|  | **ICD** | 9(60.0) | 6(40.0) |  |
|  | **CRT-D** | 11(100.0) | 0(0.0_ |  |
|  | **No** | 2352(96.4) | 89(3.6) |  |
| Lab data |  |  |  |  |
| Hemoglobin, % | **<12 or 13** | 868(93.9) | 56(6.1) | **≤0.001^b^** |
|  | **>= 12 or 13** | 1523(97.4) | 41(2.6) |  |
| Cholesterol, % | **<200** | 2134(96.0) | 89(4.0) | 0.434^b^ |
|  | **>=200** | 257(97.0) | 8(3.0) |  |
| Triglyceride, % | **<150** | 1800(95.6) | 82(4.4) | **0.037^b^** |
|  | **>=150** | 591(97.5) | 15(2.5) |  |
| HDL, % | **<50 or <40** | 1468(96.3) | 57(3.7) | 0.602^b^ |
|  | **>=50 or >=40** | 923(95.8) | 40(4.2) |  |
| LDL, % | **<130** | 2163(96.1) | 88(3.9) | 0.933^b^ |
|  | **>=130** | 228*96.2) | 9(3.8) |  |
| Na, % | **<135** | 1054(94.3) | 64(5.7) | **≤0.001^b^** |
|  | **135 to 145** | 1306(97.6) | 32(2.4) |  |
|  | **>=145** | 31(96.9) | 1(3.1) |  |
| K, % | **<3.5** | 294(97.7) | 7(2.3) | 0.191^b^ |
|  | **3.5 to 5.5** | 2004(96.0) | 84(4.0) |  |
|  | **>=5.5** | 93(93.9) | 6(6.1) |  |
| LVEF, % | **50% to 70% normal range** | 11(100.0) | 0(0.0) | **0.033^b^** |
|  | **40% to 49 mild dysfunction** | 963(97.2) | 28(2.8) |  |
|  | **30% to 39% moderate dysfunction** | 816(96.1) | 33(3.9) |  |
|  | **Less than 30% severe dysfunction** | 601(94.3) | 36(5.7) |  |
| Blood Sugar, mg/dl, Median(INQ) | | 133.00(106.00,190.00) | 146.00(110.00,226.28) | 0.117^c^ |
| WBC, per microliter, Median(INQ) | | 8.50(6.60,10.97) | 8.90(6.35,11.65) | 0.472^c^ |
| Creatinine, mg/dl, Median(INQ) | | 1.20(1.00,1.40) | 1.20(1.10,1.64) | **0.001^c^** |
| GFR, ml/min, Median(INQ) | | 54.55(40.28,70.83) | 44.19(32.01,56.83) | **≤0.001^c^** |
| Vital Signs |  |  |  |  |
| HR, % | **<60** | 215(96.0) | 9(4.0) | 0.375^b^ |
|  | **60 to100** | 1996(96.3) | 77(3.7) |  |
|  | **>100** | 180(94.2) | 11(5.8) |  |
| Systolic, mmHg, Median(INQ) | | 120.00(110.00,135.00) | 120.00(100.00,135.00) | **0.038^c^** |
| Diastolic, mmHg, Median(INQ) | | 80.00(70.00,86.00) | 75.00(62.50,80.00) | **0.003^c^** |
| Length of stay in hospital, Median(INQ) | | 3.00(2.00,5.00) | 4.00(3.00,7.50) | **0.007^c^** |
| Family History |  |  |  |  |
| Family History stroke, % | **Yes** | 158(96.9) | 5(3.1) | 0.571^b^ |
|  | **No** | 2233(96.0) | 92(4.0) |  |
| Family History heart failure, % | **Yes** | 187(97.4) | 5(2.6) | 0.335^b^ |
|  | **No** | 2204(96.0) | 92(4.0) |  |
| Family History myocardial infarction, % | **Yes** | 493(97.8) | 11(2.2) | **0.026^b^** |
|  | **No** | 1898(95.7) | 86(4.3) |  |
| Median(INQ)  Mean±SD  Number (%)  a: Independent Samples Test  b: Chi-Square  c: Mann-Whitney Test | | |  |  |

| Supplementary Table 4: characteristics of patients based on one-year mortality (N=2488) | | | | |
| --- | --- | --- | --- | --- |
|  |  | **Status (1 Year)** | | **P.value** |
|  |  | **Alive** | **Death** |  |
| Demographic information | | | | |
| Age, years, Mean±SD |  | 64.51±12.71 | 73.06±13.98 | **≤0.001^a^** |
| SEX, % | **Female** | 762(82.9) | 157(17.1) | **≤0.001^b^** |
|  | **Male** | 1384(88.2) | 185(11.8) |  |
| Ethnic, % | **Arab** | 310(88.6) | 40(11.4) | 0.296^b^ |
|  | **Fars** | 1607(85.7) | 269(14.3) |  |
|  | **Other** | 229(87.4) | 33(12.6) |  |
| Marital status, % | **Single** | 36(80.0) | 9(20.0) | 0.219^b^ |
|  | **Married** | 2110(86.4) | 333(13.6) |  |
| Body-mass index, kg/m², % | **<18.5** | 125(75.8) | 40(24.2) | **≤0.001^b^** |
|  | **18.5 to 24.9** | 1032(86.5) | 161(13.5) |  |
|  | **>=24.9** | 989(87.5) | 141(12.5) |  |
| Waist Circumference categorize, % | **<88 or 102** | 1449(86.5) | 226(13.5) | 0.598^b^ |
|  | **>=88 or 102** | 697(85.7) | 116(14.3) |  |
| Place of residence | **Fasa** | 726(85.5) | 123(14.5) | **0.049^b^** |
|  | **Other city** | 821(88.4) | 108(11.6) |  |
|  | **Village** | 599(84.4) | 111(15.6) |  |
| Drug Use |  |  |  |  |
| Smoking, % | **Yes** | 703(89.8) | 80(10.2) | **0.001^b^** |
|  | **No** | 1443(84.6) | 262(15.4) |  |
| Opium, % | **Yes** | 604(88.0) | 82(12.0) | 0.109^b^ |
|  | **No** | 1542(85.6) | 260(14.4) |  |
| Classes of Heart Failure |  |  |  |  |
| NYHA classification, % | **NYHA I** | 1104(89.0) | 136(11.0) | **≤0.001^b^** |
|  | **NYHA II** | 478(87.9) | 66(12.1) |  |
|  | **NYHA III** | 356(82.6) | 75(17.4) |  |
|  | **NYHA IV** | 208(76.2) | 65(23.8) |  |
| Underlying disease |  |  |  |  |
| Caregiver, % | **Hospital** | 2099(86.1) | 338(13.9) | 0.216^b^ |
|  | **Clinic** | 47(92.2) | 4(7.8) |  |
| Previous chronic heart failure hospitalization, % | **NO** | 1538(87.5) | 219(12.50 | **0.015^b^** |
|  | **>30 day** | 501(83.4) | 100(16.6) |  |
|  | **<30 day** | 107(82.3) | 23(17.7) |  |
| Duration of heart failure, % | **Over 6 months** | 590(82.9) | 122(17.1) | **0.002^b^** |
|  | **Less than 6 months** | 1556(87.6) | 220(12.4) |  |
| Dilated cardiomyopathy, % | **Yes** | 46(90.2) | 5(9.8) | 0.409^b^ |
|  | **No** | 2100(86.2) | 337(13.8) |  |
| RV failure, % | **Yes** | 4(100.0) | 0(0.0) | 0.424^b^ |
|  | **No** | 2142(86.2) | 342(13.8) |  |
| Previous MI, % | **Yes** | 1511(87.8) | 209(12.20 | **0.001^b^** |
|  | **No** | 635(82.7) | 133(17.3) |  |
| Atrial fibrillation/flutter, % | **Yes** | 179(81.4) | 41(18.6) | **0.027^b^** |
|  | **No** | 1967(86.7) | 301(13.3) |  |
| COPD, % | **Yes** | 69(75.0) | 23(25.0) | **0.001^b^** |
|  | **No** | 2077(86.7) | 319(13.3) |  |
| Heart valve disease, % | **Yes** | 239(84.8) | 43(15.2) | 0.437^b^ |
|  | **No** | 1907(86.4) | 299(13.6) |  |
| Previous stroke, % | **Yes** | 127(79.4) | 33(20.6) | **0.009^b^** |
|  | **No** | 2019(86.7) | 309(13.3) |  |
| Hypertension, % | **Yes** | 102(83.6) | 20(16.4) | 0.384^b^ |
|  | **No** | 2044(86.4) | 322(13.6) |  |
| Diabetes, % | **Yes** | 580(83.8) | 112(16.2) | **0.028^b^** |
|  | **No** | 1566(87.2) | 230(12.8) |  |
| Treatment |  |  |  |  |
| CABG, % | **Yes** | 272(85.3) | 47(14.7) | 0.583^b^ |
|  | **No** | 1874(86.4) | 295(13.6) |  |
| PCI, % | **Yes** | 611(88.3) | 81(11.7) | 0.067^b^ |
|  | **No** | 1535(85.5) | 261(14.5) |  |
| ECG rhythm, % | **Sinus rhythm** | 1952(87.1) | 289(12.9) | **≤0.001^b^** |
|  | **Pacemaker rhythm** | 20(71.4) | 8(28.6) |  |
|  | **Atrial fibrillation** | 14(66.7) | 7(33.3) |  |
|  | **Other** | 160(80.8) | 38(19.2) |  |
| LBBB, % | **Yes** | 242(82.0) | 53(18.0) | **0.025^b^** |
|  | **No** | 1904(86.8) | 289(13.2) |  |
| ACE Inhibitor, % | **Yes** | 1582(88.4) | 207(11.6) | **≤0.001^b^** |
|  | **No** | 564(80.7) | 135(19.3) |  |
| ARB, % | **Yes** | 427(84.4) | 79(15.6) | 0.172^b^ |
|  | **No** | 1719(86.7) | 263(13.3) |  |
| Beta blocker, % | **Yes** | 1978(87.3) | 288(12.7) | **≤0.001^b^** |
|  | **No** | 168(75.7) | 54(24.3) |  |
| Mineralocorticoid Receptor Antagonists(MRA) , % | **Yes** | 942(85.7) | 157(14.3) | 0.487^b^ |
|  | **No** | 1204(86.7) | 185(13.3) |  |
| Diuretics, % | **Yes** | 781(80.5) | 189(19.5) | **≤0.001^b^** |
|  | **No** | 1365(89.9) | 153(10.1) |  |
| Digitalis, % | **Yes** | 196(84.8) | 35(15.2) | 0.515^b^ |
|  | **No** | 1950(86.4) | 307(13.6) |  |
| Statins, % | **Yes** | 2104(86.4) | 330(13.6) | 0.067^b^ |
|  | **No** | 42(77.8) | 12(22.2) |  |
| i.v. inotropic support type, % | **Yes** | 124(62.3) | 75(37.7) | **≤0.001^b^** |
|  | **No** | 2022(88.3) | 267(11.7) |  |
| Long acting nitrates, % | **Yes** | 1199(88.7) | 153(11.3) | **≤0.001^b^** |
|  | **No** | 947(83.4) | 189(16.6) |  |
| Anticoagulants, % | **Yes** | 1858(86.0) | 302(14.0) | 0.381^b^ |
|  | **No** | 288(87.8) | 40(12.2) |  |
| ASA or Antiplatelet, % | **Yes** | 2127(86.4) | 336(13.6) | 0.135^b^ |
|  | **No** | 19(76.0) | 6(24.0) |  |
| Device Therapy, % | **Pace Maker** | 13(61.9) | 8(38.1) | **0.002^b^** |
|  | **ICD** | 15(100.0) | 0(0.0) |  |
|  | **CRT-D** | 11(100.0) | 0(0.0) |  |
|  | **No** | 2107(86.3) | 334(13.7) |  |
| Lab data |  |  |  |  |
| Hemoglobin, % | **<12 or 13** | 763(82.6) | 161(17.4) | **≤0.001^b^** |
|  | **>= 12 or 13** | 1383(88.4) | 181(11.6) |  |
| Cholesterol, % | **<200** | 1912(86.0) | 311(14.0) | 0.306^b^ |
|  | **>=200** | 234(88.3) | 31(11.7) |  |
| Trigliserid, % | **<150** | 1608(85.4) | 274(14.6) | **0.038^b^** |
|  | **>=150** | 538(88.8) | 68(11.2) |  |
| HDL, % | **<50 or <40** | 1319(86.5) | 206(13.5) | 0.665^b^ |
|  | **>=50 or >=40** | 827(85.9) | 136(14.1) |  |
| LDL, % | **<130** | 1937(86.1) | 314(13.9) | 0.364^b^ |
|  | **>=130** | 209(88.2) | 28(11.8) |  |
| Na, % | **<135** | 928(83.0) | 190(17.0) | **≤0.001^b^** |
|  | **135 to 145** | 1193(89.2) | 145(10.8) |  |
|  | **>=145** | 25(78.1) | 7(21.9) |  |
| K, % | **<3.5** | 268(89.0) | 33(11.0) | 0.218^b^ |
|  | **3.5 to 5.5** | 1796(86.0) | 292(14.0) |  |
|  | **>=5.5** | 82(82.8) | 17(17.2) |  |
| LVEF, % | **50% to 70% normal range** | 8(72.7) | 3(27.3) | **≤0.001^b^** |
|  | **40% to 49 mild dysfunction** | 889(89.7) | 102(10.3) |  |
|  | **30% to 39% moderate dysfunction** | 742(87.4) | 107(12.6) |  |
|  | **Less than 30% severe dysfunction** | 507(79.6) | 130(20.4) |  |
| Blood Sugar, mg/dl, Median(INQ) | | 132.00(105.75,187) | 150.50(113.75,231.00) | 0.117^c^ |
| WBC, per microliter, Median(INQ) | | 8.50(6.60,10.90) | 8.80(6.69,11.92) | 0.472^c^ |
| Creatinine, mg/dl, Median(INQ) | | 1.20(1.00,1.40) | 1.30(1.10,1.60) | **0.001^c^** |
| GFR, ml/min, Median(INQ) | | 55.75(41.78,71.82) | 42.14(30.15,57.48) | **≤0.001^c^** |
| Vital Signs |  |  |  |  |
| HR, % | **<60** | 187(83.5) | 37(16.5) | **≤0.001^b^** |
|  | **60 to100** | 1814(87.5) | 259(12.5) |  |
|  | **>100** | 145(75.9) | 46(24.1) |  |
| Systolic, mmHg, Median(INQ) | | 125.00(110.00,135.00) | 120.00(100.00,135.00) | **0.038^c^** |
| Diastolic, mmHg, Median(INQ) | | 80.00(70.00,87.00) | 75.00(65.00,81.00) | **0.003^c^** |
| Length of stay in hospital, Median(INQ) | | 3.00(2.00,5.00) | 4.00(2.75,6.25) | **0.007^c^** |
| Family History |  |  |  |  |
| Family History stroke, % | **Yes** | 148(90.8) | 15(9.2) | 0.081^b^ |
|  | **No** | 1998(85.9) | 327(14.1) |  |
| Family History heart failure, % | **Yes** | 177(92.2) | 15(7.8) | **0.013^b^** |
|  | **No** | 1969(85.8) | 327(14.2) |  |
| Family History myocardial infarction, % | **Yes** | 455(90.3) | 49(9.7) | **0.003^b^** |
|  | **No** | 1691(85.2) | 293(14.8) |  |
| Median(INQ)  Mean±SD  Number(%) | | |  |  |

**Supplementary Fig. 1. All stages for implementing machine learning algorithms**


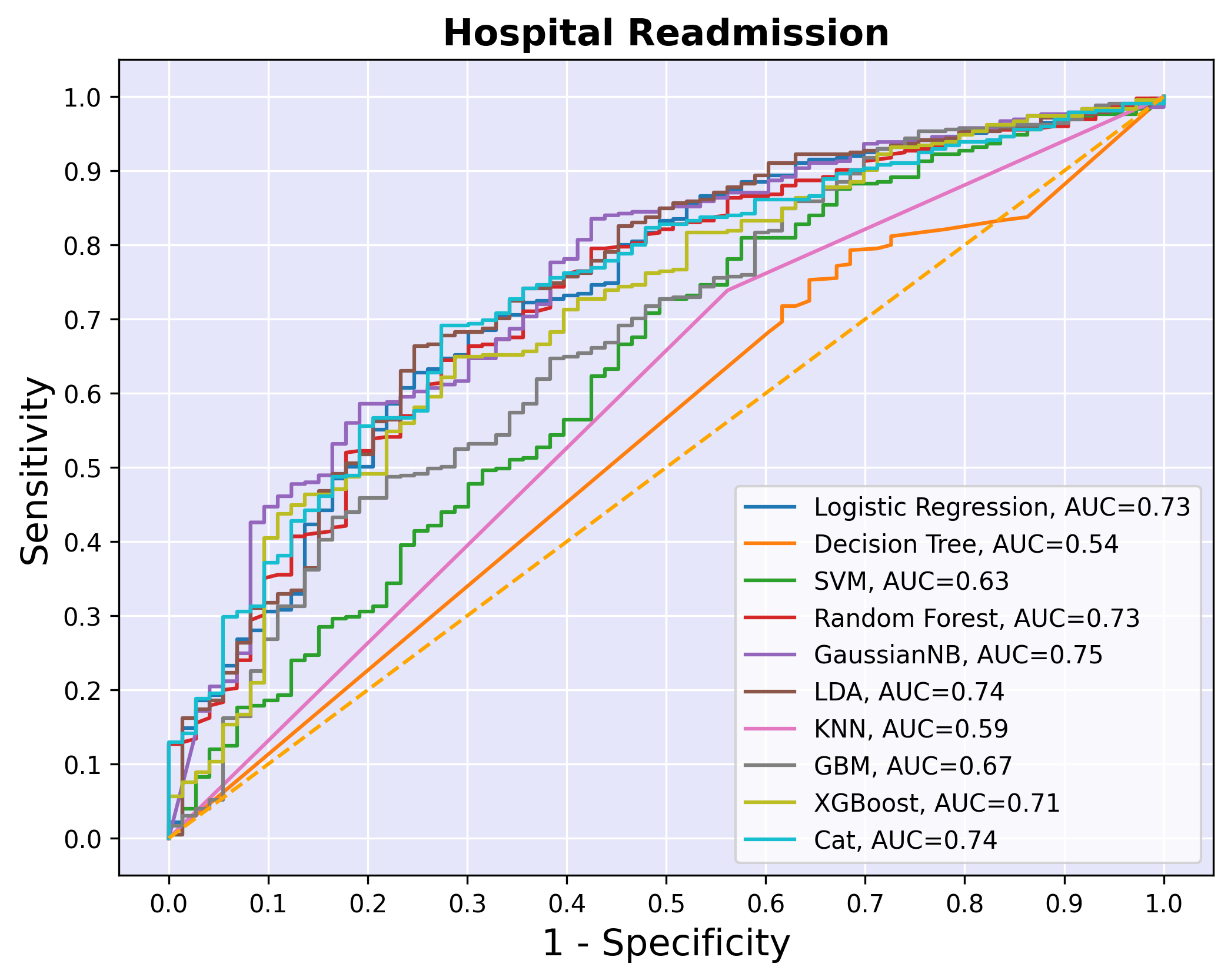

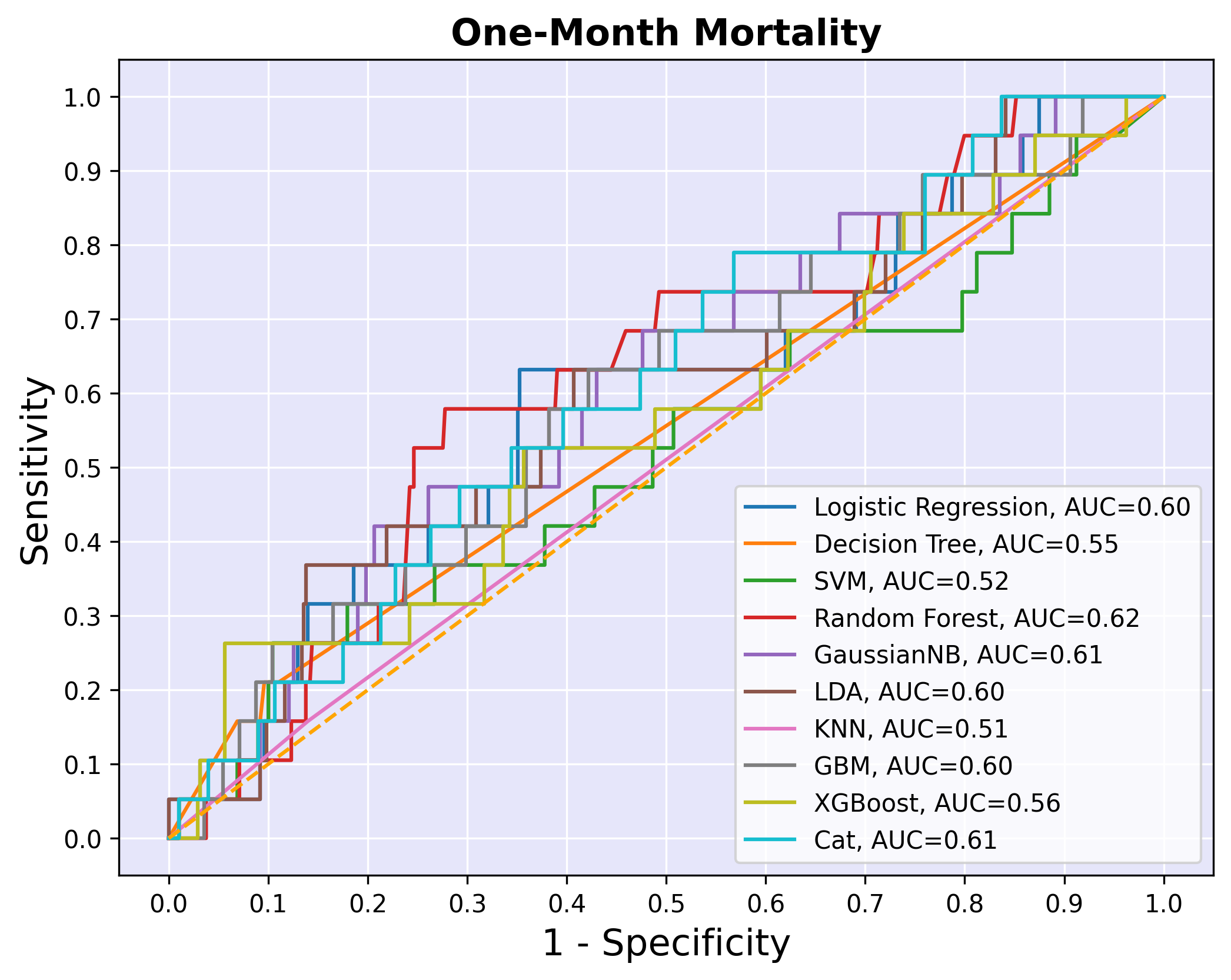

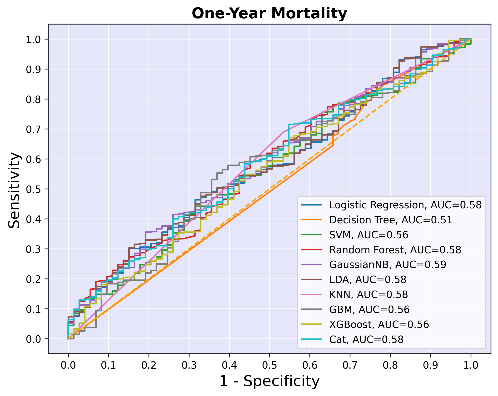


| **Supplementary Fig. 2.** The receiver operating characteristic curve among the ten models for prediction of hospital readmission, one-month mortality and one-year mortality |
| --- |
| **#Abbreviations SVM;** Support Vector Machine**, RF;** Random Forest**, Gaussian NB;** Gaussian Naive Bayes**, LDA;** Linear Discriminant Analysis**, KNN;** K-Nearest Neighbors, **GBM;** Gradient Boosting Machine, **CAT**; Cat boost. |
